# Simple Stenting Strategy with or without Branch Ostial Optimization Technique for Treatment of Coronary Bifurcation Lesions

**DOI:** 10.31083/j.rcm2306186

**Published:** 2022-05-25

**Authors:** En Chen, Wei Cai, Linlin Zhang, Lin Fan, Zhaoyang Chen, Yukun Luo, Xingchun Zheng, Chaogui Lin, Yafei Peng, Lianglong Chen

**Affiliations:** ^1^Department of Cardiology, Fujian Heart Medical Center, Fujian Medical University Union Hospital, Fujian Institute of Coronary Artery Disease, Fujian Institute of Geriatrics, 350001 Fuzhou, Fujian, China

**Keywords:** coronary bifurcation lesions, percutaneous coronary intervention, provisional stenting, proximal optimization technique

## Abstract

**Background:**

A simple stenting strategy with provisional side-branch (SB) stenting or crossover stenting has been recommended as the default approach for most coronary bifurcation lesions (CBLs). The proximal optimization technique (POT) and POT-associated techniques (POTAs) were introduced to optimize the ostium of SB. However, these techniques are unable to remove the jailed struts or completely diminish vessel damage. In this study we developed a novel branch ostial optimization technique (BOOT) and assessed its efficacy and safety by a propensity score matching comparison (PSM) with POT-associated techniques (POTA).

**Methods:**

From June 2016 to March 2018, a total of 203 consecutive patients with true CBLs were treated with BOOT (50 patients) or POTA stenting (153 patients). We performed PSM to correct for confounders from clinical and lesion characteristics. The primary endpoint was cumulative major adverse cardiac events (MACE) at 12 months including cardiac death, non-fatal myocardial infarction, and target vessel/lesion revascularization (TVR/TLR) or target vessel/lesion thrombosis (ST).

**Results:**

After PSM, there were 43 patients in each group. Follow-up coronary angiography was performed in 77 (89.5%) patients. At 12 months, the angiographic restenosis rate was significantly different between the BOOT group and the POTA group after PSM (proximal main branch: 20.01 ± 11.33% vs. 26.81 ± 14.02%, *p *= 0.003; distal main branch: 18.07 ± 3.71% vs. 23.44 ± 10.78%, *p* = 0.006; side branch: 23.53 ± 10.12% vs. 39.01 ± 10.29%, *p *< 0.001, respectively). The incidence of MACE at 12 months was not different between the BOOT group before PSM (8.0% vs. 11.8%, *p* = 0.604), but less frequent after PSM (4.7% vs. 23.3%,* p* = 0.026) when compared with the POTA group, mainly due to TVR/TLR (2.3% vs. 20.9%, *p* = 0.015).

**Conclusions:**

In patients with CBLs, BOOT is feasible for optimization of the SB ostium and may be superior to POTAs in terms of the angiographic measurements and long-term clinical outcomes at 12 months follow-up.

## 1. Introduction

Though a simple stenting strategy with provisional side-branch (SB) stenting or crossover stenting has been recommended as the default approach for most coronary bifurcation lesions (CBLs) [1, 2, 3], it is still debatable whether final kissing balloon dilation (FKBD) is necessary after main-vessel (MV) stenting [4, 5, 6, 7, 8]. Theoretically, FKBD is able to remove jailed struts and reduce ostial residual stenosis or restenosis [9, 10]. However, previous studies showed no benefits or even harm from routine FKBD [6, 7, 8]. The explanation for this discrepancy is that FKBD may damage ostial SB and deform the MV stent when removing the jailed struts [11, 12, 13]. Hence, a proximal optimization technique (POT) was introduced to facilitate restoration of fractal bifurcation anatomy, apposition of proximal struts onto the proximal MB wall, reorientation of jailing struts toward ostial SB, facilitation of distally rewiring, and partial relief of ostial SB compromise, resulting in a series of POT-associated techniques (POTAs) comprising POT-alone, SB dilation-POT (S-POT), POT-SB dilation-rePOT (POT-S-POT), kissing dilation-POT (K-POT), and POT-kissing dilation-rePOT (POT-K-POT) [1, 2, 13]. Nevertheless, these techniques remain unable to remove the jailed struts or completely diminish vessel damage [14, 15] particularly, when not rewiring SB distally [16]. We proposed a novel technique, the branch ostial optimization technique (BOOT), which can allow distal rewiring of SB and completely remove the struts across the SB ostium onto its proximal side-wall without distortion of the bifurcated vessel/stent, yielding the so-called “1-stent implantation with 2-stent effects”. Nevertheless, the efficacy of BOOT needs to be validated clinically.

This study sought to investigate whether BOOT is clinically feasible and superior to POTA when using a simple strategy for treatment of true or complex CBLs.

## 2. Materials and Methods

### 2.1 Patient’s Selection

From June 2016 to March 2018, 203 consecutive patients with true CBLs (Medina’s type 1,1,1; 0,1,1; 1,0,1) treated with a simple stenting strategy in our center were considered eligible for enrollment. Patients with ST-segment elevation acute myocardial infarction within 24 hours, life expectancy <1 year, heavy calcified anatomy, or allergy to any required drugs (aspirin, P2Y12 receptor antagonists, etc.), were excluded. The eligible patients were divided into two groups according to whether they received BOOT or POTA.

### 2.2 Procedures

**Fig. 1.**
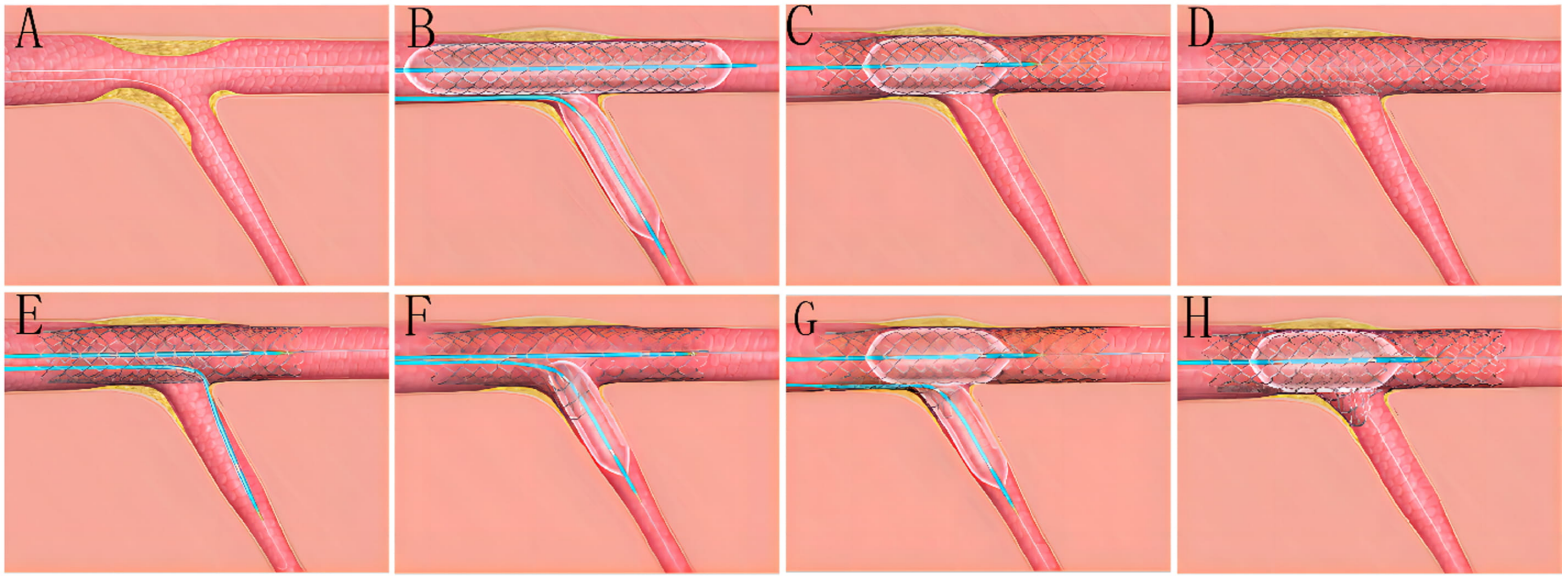
**The procedural steps of BOOT**. (A) Wiring the SB and MV and pretreating either branch as indicated. (B) Performing sequentially snuggling balloon-stent dilation (SBSD) by following steps: pre-staying a compliance balloon in the SB with its proximal maker in the bifurcation core and then properly positioning of the MV stent; first inflating the SB balloon and then stent balloon, followed by first deflating the stent balloon and then SB balloon. (C) Conducting proximal optimization technique (POT) at operator’s discretion. (D) Rewiring the SB closest to the carina. (E–G) Performing sequentially kissing or snuggling balloon dilation (SKBD/SSBD) with preferred the latter: placing 2 non-compliance balloons with mini-juxtaposition or snuggling-position in the bifurcation core and sequentially inflating the SB and MB balloons with simultaneous deflation. (H) Finalizing the procedure with (re)-POT.

BOOT: the procedural steps of BOOT include: (A) wiring the SB and MV and pretreating either branch as indicated; (B) performing sequentially snuggling balloon-stent dilation (SBSD) by the following steps: pre-staying a compliance balloon in the SB with its proximal maker in the bifurcation core and then properly positioning of the MV stent; first inflating the SB balloon and then the stent balloon, followed by first deflating the stent balloon and then SB balloon; (C) conducting the proximal optimization technique (POT) at the operator’s discretion; (D) rewiring the SB closest to the carina; (E) performing sequentially kissing or snuggling balloon dilation (SKBD/SSBD) with preference to the latter: placing 2 non-compliance balloons with mini-juxtaposition or snuggling-position in the bifurcation core and sequentially inflating the SB and MB balloons with simultaneous deflation; (F) finalizing the procedure with (re)-POT (Fig. 1).

**Fig. 2.**
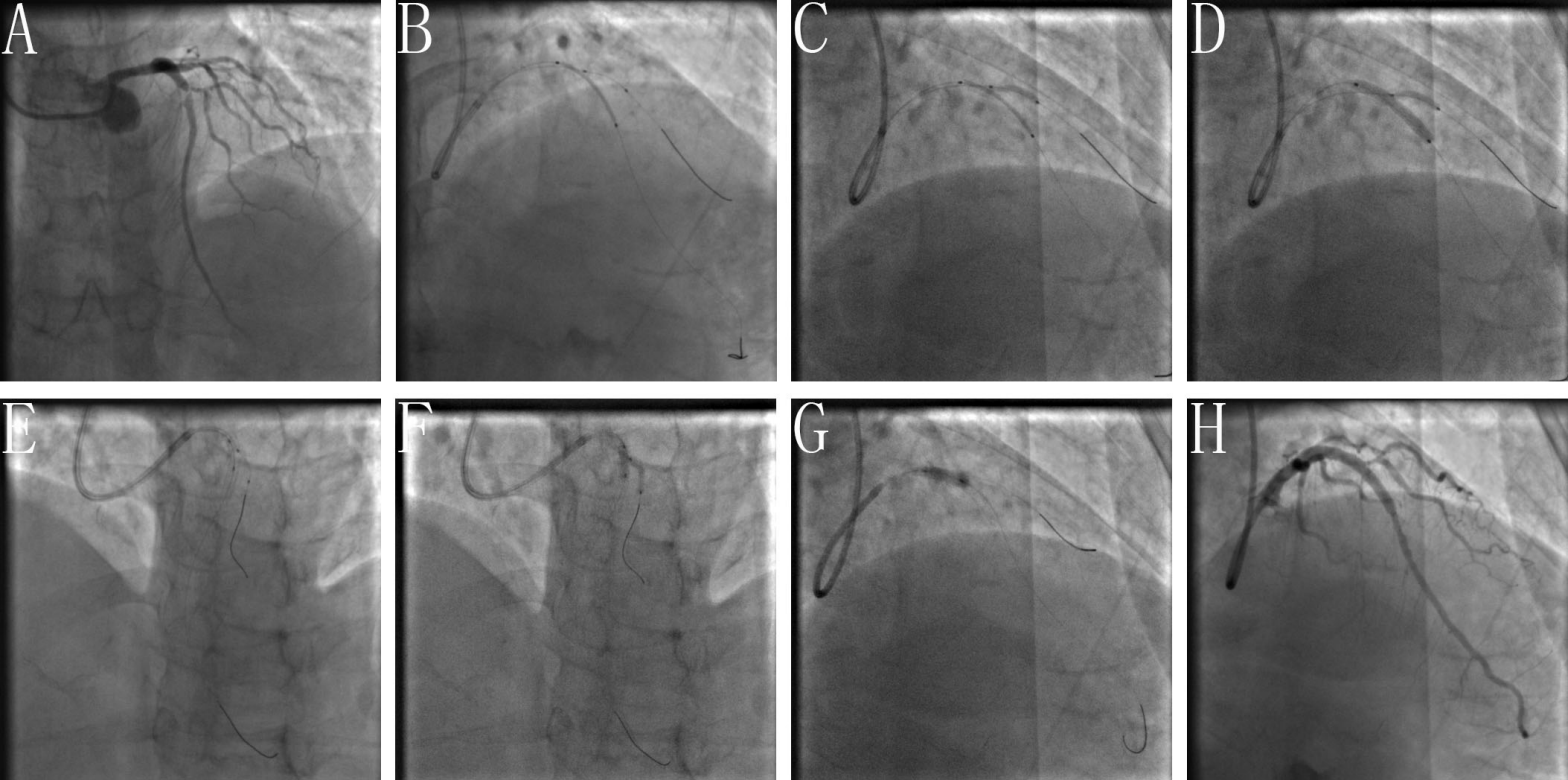
**Clinical practice of the BOOT technique**. (A) Baseline angiogram with significant stenosis of the left anterior descending (LAD)/first diagonal bifurcation (D1) (medina classification: 1, 1, 1). (B) SBSD: pre-staying a compliance balloon in the D1 with its proximal maker in the bifurcation core and then properly positioning of the LAD stent. (C) SBSD: First inflating the D1 balloon and then stent balloon, followed by first deflating the stent balloon and then D1 balloon. (D) Snuggling balloon-stent dilation. (E) After rewiring the SB closest to the carina withdraw the pre-imbedding D1 balloon and guidewire, then placing 2 non-compliance balloons with mini-juxtaposition or snuggling-position in the bifurcation core. (F) Sequentially inflating the SB and MB balloons with simultaneous deflation. (G) Finalizing the procedure with (re)-POT. (H) Final angiogram result.

**Fig. 3.**
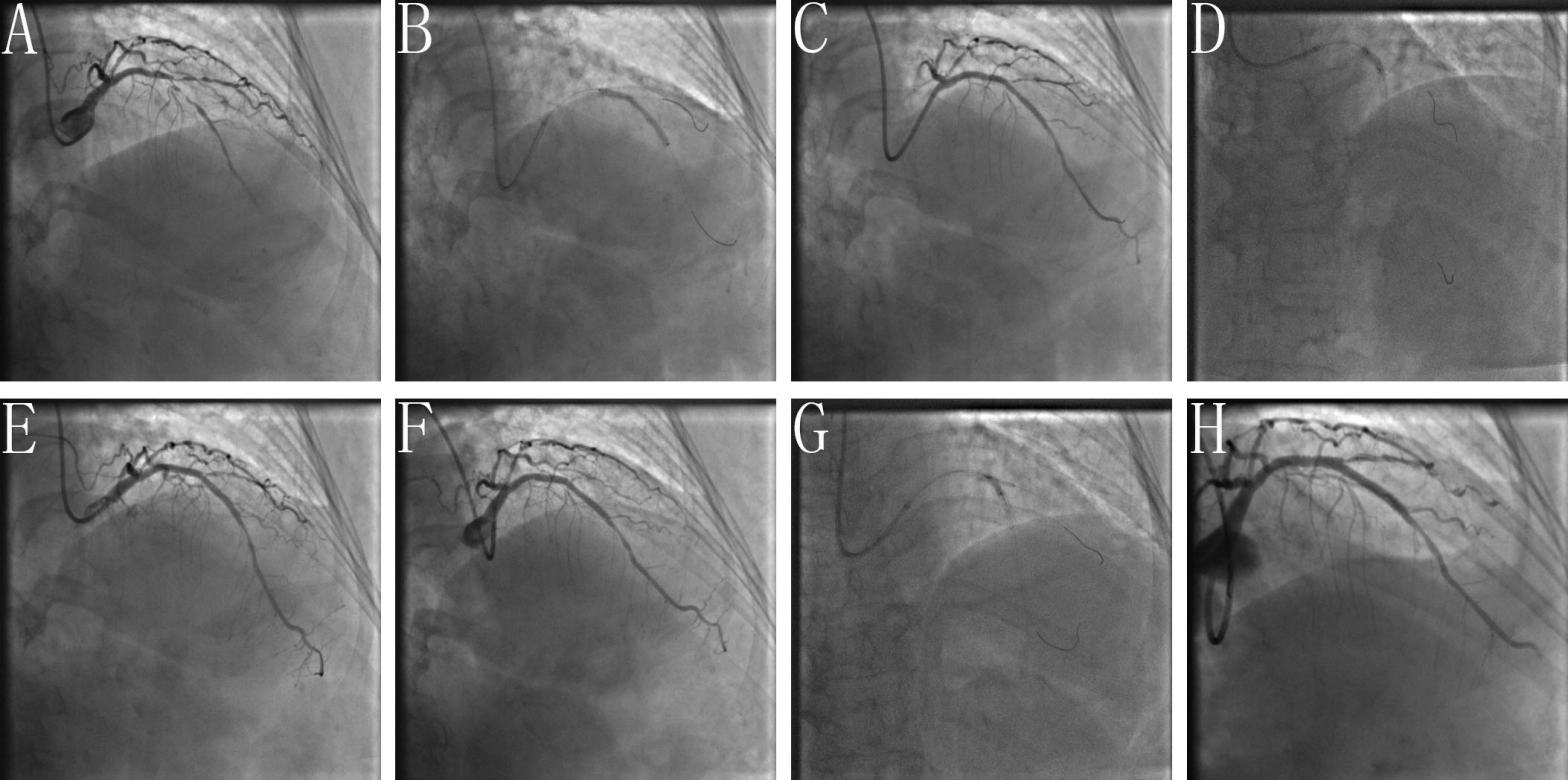
**Clinical practice of the POTA technique**. (A) Baseline angiogram with significant stenosis of the left anterior descending (LAD)/second diagonal bifurcation (D1) (medina classification: 1, 1, 1). (B) After pre-dilation of LAD and D1, the stent is positioned covering the lesion of proximal-mid LAD with pre-imbedding a guidewire to avoid the D1 acute occlusion. (C) Rewiring the D1 through the most distal cell of the LAD stent facing the SB. (D) Performing POT with NC balloon after rewiring. (E) Angiogram shown TIMI 3 blood flow of D1 with non-dissection. (F) Acute occlusion of D1 after guide wire withdrawal. (G) Performing FKBD after rewiring LAD and D1. (H) Final angiogram shown TIMI 3 blood flow of LAD with non-residual in-stent stenosis, but type B dissection of proximal D1 with TIMI 3 blood flow.

POTA: Briefly, after MV stenting, at least one of the POTAs (e.g., POT-alone, POT-S-POT, POT-K-POT) were used at the discretion of the operators. A clinical example and final results using the BOOT and POTA for treating an unstable angina patient with a severe LAD-D1 TCBL are shown by coronary angiography in Figs. 2,3, respectively.

### 2.3 Medications

Pre-procedurally, all patients received pretreatment with aspirin and the P2Y12 inhibitors clopidogrel or ticagrelor, with a loading dose as indicated. Intra-procedurally, non-fractionated heparin, 70–100 U/kg, was intravenously injected with a supplemental bolus of 1000 U given per hour to maintain an activated clotting time of 250–300 seconds. Post-procedure, aspirin was used indefinitely, and clopidogrel or ticagrelor for 12 months routinely. Peri-procedural glycoprotein IIb/IIIa inhibitors were left to the operator’s discretion.

### 2.4 Angiography Analysis

Coronary angiography was performed pre-, post-procedurally, and at follow-up after intracoronary injection of 200ug nitroglycerin, with ≥2 imaging projections with ≥30 difference. The bifurcation was segmented into three parts: (1) proximal MB, from the carina to 5-mm proximal to the end of the MV stent; (2) distal MB, from the carina to 5-mm distal to the end of the MV stent; (3) SB, 10-mm from the ostial SB.

The reference vessel diameter (RVD), minimal lumen diameter (MLD), and lesion length (LL) in 3 bifurcation segments were directly measured by QCA. The late lumen loss (LLL) was calculated by the difference between the post-procedural MLD and follow-up MLD, the diameter stenosis percent (%DS) by (RVD-MLD)/RVD × 100%. Binary restenosis was defined as %DS >50%.

### 2.5 Follow-up

Clinical data was collected during the hospital stay and by hospital visit or telephone contact at 1-, 3-, 6-, 9-, 12-months after discharge. Follow-up coronary angiography was planned at 12-month (12 ± 1 months) post-procedure.

### 2.6 Events and Definitions

The major cardiac adverse events (MACEs) were composed of cardiac death, non-fatal myocardial infarction (MI), target vessel/lesion revascularization (TVR/TLR) or target vessel/lesion thrombosis (ST). Angiographic success was defined as residual diameter stenosis <20% with grade 3 TIMI flow in both branches [17]. Additionally, severe ostial SB dissection ≥type-C according to the NHLBI classification or other requirements for bailout SB stenting was also deemed to be angiographic unsuccessful.

Non-Q-wave MI was defined by elevation of cTn values ≥5 × 99th percentile upper reference limit combined with clinical signs and without new onset of pathological Q waves; Q-wave MI as newly developing pathological Q waves in 2 contiguous leads with clinical signs. TVR/TLR was defined as the target vessel/lesion revascularization either by percutaneous coronary intervention (PCI) or coronary artery bypass grafting(CABG). ST was diagnosed according to the Academic Research Consortium definition [18].

### 2.7 Statistical Analysis

All analyses were performed with statistical software packages (SSPS 20.0, Chicago, IL, USA). Data were expressed as mean ± SD for continuous or frequency (%) for discrete or categorical variables. To compare the difference between the two groups, Student-*t* or *t*-test was employed for continuous variables, Chi-square or Fisher’s exact test for discrete ones. A *p* value < 0.05 was considered statistically significant.

Propensity score matching (PSM) was used to reduce treatment selection bias and potential impact of confounding factors from baseline clinical and lesion characteristics. Baseline clinical and lesion characteristics that could affect outcomes on univariate analysis were deemed as candidate variables. The reliability of the model was evaluated using the Hosmer-Lemeshow test. Based on the nearest match algorithm, we created case-matched pairs without replacement at a ratio of 1:1.

## 3. Results

Among 203 eligible patients, 50 received BOOT and 153 received POTA, among them 43 pairs of patients were matched for baseline clinical and lesion characteristics by PSM. Matching was performed, based on age, hypertension, diabetes, RVD of proximal and distal main branch (MB), diameter stenosis of proximal and distal MB, LL of proximal and distal MB, RVD, diameter stenosis and LL of SB, with a caliper width equal to 0.1. Before PSM, 46 patients of the BOOT group and 134 patients of the POTA group underwent angiographic follow-up, respectively. After PSM, there were 39 patients of the BOOT group and 38 patients of the POTA group who underwent angiographic follow-up, respectively. Except for shorter lesion length and less diameter stenosis of the SB in the BOOT group before PSM, there were no differences in the baseline clinical and lesion characteristics or in the MV stent length and number between the two groups before or after PSM (Table 1).

**Table 1.** **Baseline clinical characteristics**.

		Before PSM			After PSM		
		BOOT (n = 50)	POTA (n = 153)	*p *values	BOOT (n = 43)	POTA (n = 43)	*p* values
Male, n (%)		47 (94.0)	127 (83.0)	0.054	40 (93.0)	39 (90.7)	1.000
Age (years)		63.5 ± 9.9	66.2 ± 10.8	0.115	63.4 ± 9.7	61.9 ± 11.1	0.522
Hypertension, n (%)		34 (68.0)	91 (59.5)	0.282	28 (55.1)	34 (79.1)	0.149
Hypercholesterolemia, n (%)		26 (52.0)	87 (56.9)	0.548	16 (37.2)	18 (41.9)	0.659
Diabetes, n (%)		14 (28.0)	51 (33.6)	0.466	13 (30.2)	18 (41.9)	0.261
Smoking, n (%)		25 (50.0)	76 (49.7)	0.968	21 (48.8)	19 (43.2)	0.597
Prior PCI, n (%)		8 (16.0)	33 (21.6)	0.394	6 (14.0)	6 (14.0)	1.000
Prior MI, n (%)		7 (14.0)	22 (14.4)	0.947	4 (9.3)	8 (18.6)	0.351
LVEF (%)		60.7 ± 1 0.3	60.4 ± 12.4	0.86	61.4 ± 9.4	63.2 ± 9.3	0.366
Coronary artery disease, n (%)							
	Stable angina pectoris	27 (54.0)	95 (62.1)	0.310	27 (62.8)	29 (67.4)	0.651
	Unstablem angina pectoris	17 (34.0)	39 (25.5)	0.242	12 (27.9)	11 (25.6)	0.808
	NSTEMI	6 (12.0)	19 (12.4)	0.938	4 (9.3)	3 (7.0)	1.000
Antiplatelet therapy, n (%)							
	Aspirin	50 (100)	153 (100)	1.000	43 (100)	43 (100)	1.000
	Clopidogrel/Ticargrelor	50 (100)	153 (100)	1.000	43 (100)	43 (100)	1.000
	GP IIb/IIIa inhibitors	8 (16.0)	25 (16.3)	0.955	5 (11.6)	4 (9.3)	1.000

Data are presented as mean ± SD or number (percentages). Abbreviations: BOOT, branch ostial optimization technique; POTA, POT-associated technique; PSM, propensity score matching; MI, myocardial infarction; PCI, percutaneous coronary intervention; NSTEMI, non-ST elevation MI; LVEF, left ventricular ejection fraction.

### 3.1 Procedural Data

Before PSM, angiographic success was significantly higher in the BOOT group than in the POTA group (78.0% vs. 47.1%, *p *< 0.001), which was mostly due to a significant reduction of ≥20% residual ostial stenosis (24.0% vs. 61.4%, *p *< 0.001), partly due to a reduction of ≤3 TIMI flow (2.0% vs. 9.8%, *p* = 0.126), ≥Type-C dissection (4.0% vs. 7.8%, *p* = 0.525) and bailout stenting (4.0% vs. 11.1%, *p* = 0.169) in the SB. After PSM, angiographic success remained significantly higher in the BOOT group than in the POTA group, which was also due to a significant reduction of ≥20% residual ostial stenosis (25.6% vs. 60.5%, *p* = 0.001), ≤3TIMI flow (0% vs. 20.9%, *p* = 0.002), and partially due to a reduction of ≥Type-C dissection (2.3% vs. 15.9%, *p* = 0.058) and bailout stenting (2.3% vs. 15.9%, *p* = 0.058) in the SB (Table 2).

**Table 2.** **Lesion’s and procedural characteristics**.

		Before PSM			After PSM		
		BOOT (n = 50)	POTA (n = 153)	*p* values	BOOT (n = 43)	POTA (n = 43)	*p* values
Lesion locations, n (%)				0.145			0.289
	LM-CBLs	12 (24.0)	23 (15.0)		7 (16.3)	11 (25.6)	
	Non-LM-CBLs	38 (76.0)	130 (85.0)		36 (83.7)	32 (74.4)	
Lesion length (mm)							
	Proximal MB	15.82 ± 10.46	13.28 ± 9.61	0.114	14.55 ± 9.99	16.52 ± 12.07	0.413
	Distal MB	16.09 ± 5.62	15.66 ± 5.31	0.630	15.72 ± 5.32	16.63 ± 5.56	0.437
	SB	10.52 ± 2.20	12.64 ± 2.25	0.014	12.35 ± 2.10	12.21 ± 2.58	0.797
Reference vessel diameter (mm)							
	Proximal MB	3.41 ± 0.37	3.32 ± 0.26	0.098	3.40 ± 0.36	3.39 ± 0.28	0.854
	Distal MB	2.74 ± 0.33	2.64 ± 0.22	0.052	2.96 ± 0.33	2.93 ± 0.23	0.994
	SB	2.30 ± 0.25	2.26 ± 0.27	0.361	2.29 ± 0.25	2.27 ± 0.29	0.751
Diameter stenosis (%)							
	Proximal MB	78.17 ± 7.26	79.58 ± 5.41	0.145	79.09 ± 6.97	77.69 ± 5.73	0.310
	Distal MB	80.33 ± 6.97	78.75 ± 5.98	0.121	80.52 ± 7.47	79.19 ± 6.41	0.379
	SB	74.41 ± 6.79	76.58 ± 5.93	0.031	75.04 ± 7.01	76.64 ± 6.22	0.268
	POT, n (%)	50 (100)	148 (96.7)	0.442	43 (100)	43 (100)	1.000
MV stenting							
	Stent length (mm)	33.44 ± 13.30	31.20 ± 12.86	0.291	31.86 ± 12.01	35.56 ± 15.52	0.220
	Stent numbers (n)	1.24 ± 0.43	1.18 ± 0.39	0.382	1.19 ± 0.39	1.30 ± 0.46	0.214
Residual stenosis ≥20%, n (%)							
	MB	0 (0.0)	0 (0.0)	1.000	0 (0.0)	0 (0.0)	1.000
	SB	12 (24.0)	94 (61.4)	<0.001	11 (25.6)	26 (60.5)	0.001
TIMI flow ≤3, n (%)							
	MB	0 (0.0)	0 (0.0)	1.000	0 (0.0)	0 (0.0)	1.000
	SB	1 (2.0)	15 (9.8)	0.126	0 (0.0)	9 (20.9)	0.002
	SB dissection ≥type C, n (%)	2 (4.0)	12 (7.8)	0.525	1 (2.3)	7 (15.9)	0.058
	SB bailout stenting*, n (%)	2 (4.0)	17 (11.1)	0.169	1 (2.3)	7 (15.9)	0.058
	SB angiographic success, n (%)	39 (78.0)	72 (47.1)	<0.001	31 (72.1)	12 (27.9)	0.005
	Procedural time (min)	31.22 ± 1.91	30.70 ± 2.59	0.194	31.24 ± 1.80	31.56 ± 2.94	0.552
	Fluoroscopy time (min)	20.64 ± 1.62	20.28 ± 2.19	0.286	20.67 ± 1.61	21.20 ± 2.03	0.184
	Contrast volume (mL)	129.44 ± 7.50	126.69 ± 5.48	0.020	128.84 ± 7.49	127.63 ± 5.87	0.407

Data are presented as mean ± SD or number (percentages). Abbreviations: LM-CBLs, left main coronary bifurcation lesions; MB, main-branch; MV, main-vessel; POT, proximal optimization technique; SB, side-branch; TIMI, thrombolysis in myocardial infarction. * Bailout stenting was indicated only when TIMI flow ≤3 or dissection ≥type C.

### 3.2 QCA Data

At baseline, RVD, MLD, %DS and LL were comparable in all segments of the proximal MB, distal MB and SB between the groups; immediately post-procedure, comparing BOOT versus POTA groups, there was larger MLD and less %DS in each bifurcated segment, particularly in SB (MLD: 2.10 ± 0.29 mm vs. 1.90 ± 0.22 mm, *p* = 0.031; %DS: 14.00 ± 8.13% vs. 18.61 ± 6.36%, *p* = 0.004) at 12 months follow-up. When comparing BOOT versus POTA groups, there remained larger MLD and less %DS along with less LLL and lower restenosis rate in each bifurcated segment, particularly in SB (MLD: 1.84 ± 0.30 mm vs. 1.48 ± 0.32 mm, *p *< 0.001; %DS: 23.53 ± 10.12% vs. 39.01 ± 10.29%, *p *< 0.001; LLL: 0.16 ± 0.14 mm vs. 0.43 ± 0.28 mm, *p *< 0.001; restenosis rate: 2.6% vs. 23.7%, *p* = 0.007) (Table 3).

**Table 3.** **QCA measurements between two treatments after propensity score matching**.

		Proximal MB			Distal MB			SB		
		BOOT	POTA	*p* values	BOOT	POTA	*p* values	BOOT	POTA	*p* values
		(n = 39)	(n = 38)		(n = 39)	(n = 38)		(n = 39)	(n = 38)	
Baseline										
	RVD (mm)	3.40 ± 0.36	3.39 ± 0.28	0.854	2.96 ± 0.33	2.93 ± 0.23	0.994	2.29 ± 0.25	2.27 ± 0.29	0.751
	MLD (mm)	0.71 ± 0.23	0.76 ± 0.22	0.273	0.60 ± 0.21	0.61 ± 0.18	0.365	0.57 ± 0.17	0.53 ± 0.15	0.203
	%DS	79.09 ± 6.97	77.69 ± 5.73	0.310	80.52 ± 7.47	79.19 ± 6.41	0.379	75.04 ± 7.01	76.64 ± 6.22	0.268
	LL (mm)	14.55 ± 9.99	16.52 ± 12.07	0.413	15.72 ± 5.32	16.63 ± 5.56	0.437	12.35 ± 2.10	12.21 ± 2.58	0.797
Post-procedure										
	RVD (mm)	3.41 ± 0.30	3.38 ± 0.29	0.812	2.97 ± 0.34	2.95 ± 0.24	0.73	2.32 ± 0.24	2.34 ± 0.28	0.820
	MLD (mm)	2.89 ± 0.35	2.80 ± 0.31	0.722	2.73 ± 0.30	2.64 ± 0.23	0.156	2.10 ± 0.29	1.90 ± 0.22	0.031
	%DS	16.24 ± 2.31	18.98 ± 2.33	0.011	8.11 ± 1.93	10.33 ± 1.39	<0.001	14.00 ± 8.13	18.61 ± 6.36	0.004
Follow-up										
	RVD (mm)	3.45 ± 0.37	3.43 ± 0.33	0.672	3.01 ± 0.34	2.94 ± 0.26	0.372	2.42 ± 0.24	2.42 ± 0.30	0.954
	MLD (mm)	2.78 ± 0.32	2.57 ± 0.34	0.029	2.46 ± 0.30	2.26 ± 0.39	0.012	1.84 ± 0.30	1.48 ± 0.32	<0.001
	%DS	20.01 ± 11.33	26.81 ± 14.02	0.003	18.07 ± 3.71	23.44 ± 10.78	0.006	23.53 ± 10.12	39.01 ± 10.29	<0.001
	LLL (mm)	0.13 ± 0.10	0.22 ± 0.15	0.040	0.26 ± 0.12	0.37 ± 0.32	0.050	0.16 ± 0.14	0.43 ± 0.28	<0.001
	Restenosis rate, n (%)	0 (0.0)	3 (7.9)	0.115	0 (0.0)	3 (7.9)	0.115	1 (2.6)	9 (23.7)	0.007

Data are presented as mean ± SD or number (percentages). Abbreviations: DS, diameter stenosis; LL, lesion length; LLL, late lumen loss; MLD, minimal lumen diameter; MV, main-branch; RVD, reference vessel diameter; SB, side-branch. * Before PSM, 46 patients of the BOOT group and 134 patients of the POTA group underwent angiographic follow-up, respectively. After PSM, there were 39 patients of the BOOT group and 38 patients of the POTA group who underwent angiographic follow-up, respectively.

### 3.3 Clinical Outcomes

During hospitalization, MACE was rare and similar between BOOT and POTA groups before PSM (2.0% vs. 3.9%,* p* = 1.000) and after PSM (0.0% vs. 4.7%, *p* = 0.494). At 12 months follow-up, MACE was similar before PSM (8.0% vs. 11.8%, *p* = 0.604) between the groups and less frequent after PSM (4.7% vs. 23.3%, *p* = 0.026) in the BOOT group than in the POTA group, due mainly to TVR/TLR (2.3% vs. 20.9%, *p* = 0.015) (Table 4).

**Table 4.** **QCA measurements between two treatments after propensity score matching**.

		Before propensity score matching			After propensity score matching		
		BOOT (n = 50)	POTA (n = 153)	*p* values	BOOT (n = 43)	POTA (n = 43)	*p* values
MACE in hospital, n (%)		1.000 (2.0)	6 (3.9)	1.000	0 (0.0)	2 (4.7)	0.494
	Non-Cardiac death, n (%)	0 (0.0)	0 (0.0)	1.000	0 (0.0)	0 (0.0)	1.000
	Cardiac death, n (%)	0 (0.0)	0 (0.0)	1.000	0 (0.0)	0 (0.0)	1.000
	Non-Q-wave MI, n (%)	1 (2.0)	6 (3.9)	1.000	0 (0.0)	2 (4.7)	0.494
	Q-wave MI, n (%)	0 (0.0)	0 (0.0)	1.000	0 (0.0)	0 (0.0)	1.000
	Stent thrombosis, n (%)	0 (0.0)	0 (0.0)	1.000	0 (0.0)	0 (0.0)	1.000
	Urgent TVR/TLR, n (%)	0 (0.0)	0 (0.0)	1.000	0 (0.0)	0 (0.0)	1.000
MACE at follow-up, n (%)		4 (8.0)	18 (11.8)	0.604	2 (4.7)	10 (23.3)	0.026
	Non-cardiac death, n (%)	0 (0.0)	0 (0.0)	1.000	0 (0.0)	0 (0.0)	1.000
	Cardiac death, n (%)	0 (0.0)	0 (0.0)	1.000	0 (0.0)	0 (0.0)	1.000
	Non-Q-wave MI, n (%)	1 (2.0)	2 (1.3)	1.000	1 (2.3)	0 (0.0)	1.000
	Q-wave MI, n (%)	0 (0.0)	1 (0.7)	1.000	0 (0.0)	1 (2.3)	1.000
	Stent thrombosis, n (%)	1 (2.0)	3 (2.0)	1.000	1 (2.3)	1 (2.3)	1.000
	TVR/TLR, n (%)	2 (4.0)	13 (8.5)	0.368	1.000 (2.3)	9 (20.9)	0.015

Abbreviations: MACE, major adverse cardiovascular event; MI, myocardial infarction; TVR/TLR, target vessel/ lesion revascularization.

## 4. Discussion

An optimal treatment of a pinched SB is still in debate when using a simple stenting strategy [1]. This study compared POTA versus BOOT for optimization of ostial SB in treatment of true or complex CBLs with provisional SB stenting or crossover stenting. Our major findings were (1) BOOT significantly improved immediate angiographic success by reducing residual stenosis, abnormal TIMI flow, severe dissection and bailout stenting of SB; (2) BOOT significantly reduced MLD, LLL, %DS and restenosis rate at 1-year angiographic follow-up in each bifurcated segment especially in SB; (3) BOOT also significantly reduced cumulative MACE mainly by reducing TVR/TLR.

### 4.1 BOOT Versus POTA

When using a simple stenting strategy for treatment of CBLs, routine FKBD is inadvisable due to its undesirable effects and inconsistent clinical outcomes [4, 5, 6, 7, 8, 19, 20, 21]; whereas POT is recommended because of its technically simplicity, but also multiply benefits, such as restoration of fractal bifurcation anatomy, apposition of proximal struts onto the proximal MB wall, prevention of wrong-way wiring, reorientation of jailing struts toward the ostial SB, facilitation of distally rewiring, and partial relief of ostial SB compromise [1, 2, 13, 16, 22]. Nevertheless, POT-alone can only partially relieve ostial SB compromise because it provides only reorientation but not complete apposition of the jailing struts onto the proximal side-wall of ostial SB, likely leaving the struts jailed in the midportion of the SB ostium [14, 15]. A recent multicenter registry investigated the efficacy of POT on crossover stenting under optical coherence tomography (OCT) guidance and showed that pre-POT (POT before MV stenting) provided no benefits such as reduction of incomplete strut apposition around the bifurcation or no increased success of guide wire re-crossing into the optimal cell [23]. In addition to POT-alone, POT can be used before or/and after FKBD or isolated SB dilation in different sequences, resulting in several combinations of POTAs (e.g., S-POT, K-POT, POT-S-rePOT, POT-K-rePOT), all of which, especially the re-POT, have been well accepted and recommended by the 11th and 12th consensuses of the European Bifurcation Club [1, 2]. However, despite the fact that S-POT and K-POT can more fully open the ostial SB, they may also cause problematic deformation of the MV stent or/and the MV itself in and beyond the bifurcation core [1, 2, 13], which can be corrected by adding a final POT to S-POT/K-POT (POT-S-rePOT or POT-K-rePOT), indicating that the re-POT is a crucial step of POTAs [13, 24].

Nevertheless, the actual results of POT-K-rePOT and POT-S-rePOT were still questioned. Our previous study showed that despite well-apposition of the struts onto the proximal vascular wall of a bifurcation, POTAs remained unable to completely remove the ostial jailed struts, even after adding a final re-POT. Conversely we also noted that displacing the jailing struts by FKBD or SB dilation frequently turned back after a final POT, leaving the struts jailed in the mid-portion of the ostial SB [14, 15]. Additionally, several previous studies also found detrimental effects due to the proximal overstretch induced by simultaneous kissing inflation of juxtaposing balloons [19, 20, 21]. Finally, currently, there are no large scale randomized clinical trials to confirm the clinical efficacy of POTAs [1, 2].

Unlike POTAs, BOOT, as shown in our previous bench testing [14, 15], is characterized by 2 crucial steps of SBSD and SKBD/SSBD (Fig. 1), SBSD enables us to distally rewire the SB closer to the carina (a key prerequisite for subsequent high quality BOOT) because it can actively prevent carina and/or plaque shifting; and SKBD/SSBD can effectively displace the jailing struts opposing onto the proximal side-wall of the SB ostium without inducing stent distortion and luminal asymmetry in the bifurcation core, resulting in the so called “lip-like ectropion of ostial struts” or “1-stent implantation with 2-stent effects”. Such favorable results observed in bench testing can also be translated into the improvement of the immediate angiographic success, follow-up angiographic results, and clinical outcomes at 1-year follow-up as demonstrated in our study.

### 4.2 Clinical Relevance of BOOT

As we have noted, POTAs were not powerful enough to correct SB compromise and MV stent distortion. A pinched SB may cause myocardial ischemia and will affect intra-procedural passage of devices (drug-coated balloon, IVUS, OCT etc.) or future SB-downstream lesion intervention. Conversely, BOOT, by its ability to fully open ostial SB without extra damage of the bifurcated vessel and/or stent, will benefit PSS or crossover stenting in several aspects: (1) affording an active protection to prevent intra-procedural SB occlusion by SSBD, securing safe application of simple stenting techniques as the initial strategy for CBLs; (2) facilitating distal rewiring of SB by SBSD, thereby avoiding intra-procedural use of OCT for guidance of distal rewiring; (3) efficiently displacing and opposing the jailing struts onto the proximal side-wall of the ostial SB by SKBD/SSBD. Overall, BOOT enables us to effectively optimize ostial SB, to finally achieve the goal of “1-stent implantation with 2-stent effects” in the majority of clinical situations, ultimately avoiding complex 2-stent techniques.

### 4.3 Limitations

Although PSM was used to reduce selection and treatment bias and potential cofounders that may impact clinical outcomes, our study still had several limitations. First, this study was an observational, single-center study with a limited sample size. Second, pre-staying a balloon in the SB and performing SSBD may induce a potential risk of damaging the stent polymer layer or the stent itself. Third, the procedural steps may be unfamaliar for inexperienced operators. Fourth, due to the inadequate power of clinical endpoints, the conclusions of our study should be interpreted with caution. Therefore, we are conducting a randomized clinical study to confirm our observations.

## 5. Conclusions

When using a simple stenting strategy for CBLs, BOOT is feasible for optimization of the SB ostium and may be superior to POTAs in terms of the immediate angiographic success, QCA measurements and long-term clinical outcomes at one-year follow-up. Randomized clinical studies will be required to further validate our findings. When using a simple stenting strategy for CBLs, BOOT is feasible for optimization of the SB ostium and may be superior to POTAs in terms of the immediate angiographic success, QCA measurements and long-term clinical outcomes at one-year follow-up. Randomized clinical studies will be necessary to further validate our findings.

## Abbreviations

SB, side-branch; CBLs, coronary bifurcation lesions; FKBD, final kissing balloon dilation; MV, main-vessel; POT, proximal optimization technique; POTAs, POT-associated techniques; S-POT, SB dilation-POT; POT-S-POT, POT-SB dilation-rePOT; K-POT, kissing dilation-POT; POT-K-POT, POT-kissing dilation-rePOT; BOOT, branch ostial optimization technique; SBSD, sequentially snuggling balloon-stent dilation; SKBD/SSBD, sequentially kissing or snuggling balloon dilation; RVD, reference vessel diameter; MLD, minimal lumen diameter; LL, lesion length; LLL, late lumen loss; %DS, diameter stenosis percent; MACEs, major cardiac adverse events; MI, non-fatal myocardial infarction; TVR/TLR, target vessel/lesion revascularization; ST, target vessel/lesion thrombosis; PCI, percutaneous coronary intervention; CABG, coronary artery bypass grafting; PSM, propensity score matching; MB, main branch; OCT, optical coherence tomography.
